# Unprecedented strong and reversible atomic orbital hybridization enables a highly stable Li–S battery

**DOI:** 10.1093/nsr/nwac078

**Published:** 2022-04-21

**Authors:** Min Yan, Wenda Dong, Fu Liu, Lihua Chen, Tawfique Hasan, Yu Li, Bao-Lian Su

**Affiliations:** State Key Laboratory of Advanced Technology for Materials Synthesis and Processing, Wuhan University of Technology, Wuhan 430070, China; Hubei Key Laboratory of Plasma Chemistry and Advanced Materials, Hubei Engineering Technology Research Center of Optoelectronic and New Energy Materials, School of Materials Science and Engineering, Wuhan Institute of Technology, Wuhan 430205, China; State Key Laboratory of Advanced Technology for Materials Synthesis and Processing, Wuhan University of Technology, Wuhan 430070, China; School of Materials Science and Engineering, Zhejiang University, Hangzhou 310027, China; State Key Laboratory of Advanced Technology for Materials Synthesis and Processing, Wuhan University of Technology, Wuhan 430070, China; Cambridge Graphene Centre, University of Cambridge, Cambridge CB3 0FA, UK; State Key Laboratory of Advanced Technology for Materials Synthesis and Processing, Wuhan University of Technology, Wuhan 430070, China; State Key Laboratory of Advanced Technology for Materials Synthesis and Processing, Wuhan University of Technology, Wuhan 430070, China; Laboratory of Inorganic Materials Chemistry (CMI), University of Namur, Namur B-5000, Belgium

**Keywords:** atomic orbital hybridization, hierarachical hollow sandwiched sulfur nanosphere, double sulfur–polyaniline networks, lithium–sulfur batteries, *i* *n* *situ* vulcanization

## Abstract

The shuttle effect and excessive volume change of the sulfur cathode severely impede the industrial implementation of Li–S batteries. It is still highly challenging to find an efficient way to suppress the shuttle effect and volume expansion. Here, we report, for the first time, an innovative atomic orbital hybridization concept to construct the hierarchical hollow sandwiched sulfur nanospheres with double-polyaniline layers as the cathode material for large-scale high-performance Li–S batteries. This hierarchically 3D, cross-linked and stable sulfur–polyaniline backbone with interconnected disulfide bonds provides a new type and strong intrinsic chemical confinement of sulfur owing to the atomic orbital hybridization of Li 2s, S 3p, C 2p and N 2p. Crucially, such atomic orbital hybridization of sulfur sandwiched in the double sulfur–polyaniline network is highly reversible during the discharge/charge process and can very efficiently suppress the shuttle effect and volume expansion, contributing to a very high capacity of 1142 mAh g^–1^ and an excellent stabilized capacity of 886 mAh g^–1^ at 0.2 C after 500 cycles with a suppressed volume expansion and an unprecedented electrode integrity. This innovative atomic orbital hybridization concept can be extended to the preparation of other electrode materials to eliminate the shuttle effect and volume expansion in battery technologies. The present work also provides a commercially viable and up-scalable cathode material based on this strong and highly reversible atomic orbital hybridation for large-scale high-performance Li–S batteries.

## Teaser

A hollow sandwiched sulfur nanospheres with a double S-PANI network offering strong structural and chemical confinement via atomic orbital hybridization exhibits a high initial capacity, long-term stability and electrode integrity.

## INTRODUCTION

Lithium–sulfur (Li–S) batteries, based on naturally abundant, non-toxic and environmentally benign sulfur, having a high theoretical capacity of 1675 mAh g^–1^ and an energy density of 2600 Wh kg^–1^, show great potential to be the future of energy-storage systems [1–4]. However, the commercialization of sulfur-based cathodes for Li–S batteries, in spite of intensive research efforts, still faces several important obstacles. These include the shuttle effect due to the dissolution of intermediate lithium polysulfides (LiPSs) in the liquid electrolyte, the electrically insulating nature of sulfur (∼5 × 10^–30^ S cm^–1^ at 25°C) and the large (∼76%) volume change that induces severe disintegration of the electrodes in the delithiation/lithiation process [5,6]. All these phenomena are strongly detrimental to the charge/discharge capacity and the long-term cycling performance, and remain the most critical hurdles towards the commercial production of Li–S batteries [7,8].

Conducting polymers, such as polythiophene and polydopamine, have been widely explored as matrices to physically confine sulfur and provide essential electrical contact through their organic framework [9–11]. Although the electrical conductivity was improved, such physical confinement was insufficient to prevent the dissolution of LiPSs [12,13]. Subsequently chemical interactions via polar bonding were introduced into sulfur–polymer composites to trap LiPSs under subtler scales [9,14–16]. In addition, the accommodation of volume expansion using an optimized structure promoted cycle stability [17–19]. For example, porous [20,21], core–shell [22,23] and hollow spheres [24–26] were shown to relieve the stress derived from volume expansion during cycling and to facilitate high energy density. All these approaches have contributed to great progress in the cycling stability, cycle life and specific capacity of Li–S batteries [27–30]. However, due to the sophisticated and tedious preparation process and the use of costly and/or environmentally hazardous raw materials that limit the fabrication of the materials to small batches, these approaches are not commercially viable. Furthermore, the safety issues concerning materials processing and fabrication employed in these strategies also require consideration. The development of a commercially viable strategy for the synthesis of functional cathode materials towards large-scale and safe Li–S batteries to simultaneously suppress shuttle effect and volume expansion with long-term electrochemical performance and stability remains highly challenging.

Herein, we report an upscalable and potentially the first commercially viable process for Li–S cathode materials. We designed hierarchical hollow sandwiched sulfur nanospheres (HNPs) with double-polyaniline (PANI) layers (S–PANI@S@S–PANI–HNPs) to strongly anchor the sulfur species. The *insitu* vulcanization of the PANI@S@ PANI–HNPs led to the reaction of the elemental sulfur with the PANI to form a cross-linked, structurally stable sulfur–polyaniline (S–PANI) polymer network with interconnected disulfide bonds that provided synergistically strong structural and chemical double confinement for the sulfur species. We report, for the first time, a new type of intrinsic chemical confinement via a highly reversible atomic orbital hybridization between Li 2s, S 3p, C 2p and N 2p that leads to the strong but reversible chemical bonding of S–PANI to LiPSs. Our new strategy to develop highly reversible chemically linked sulfur in the S–PANI polymer network, in addition to the sandwich structural effect, offers high sulfur utilization and a very high rate of transport of e^–^ and Li^+^ from inside the hollow structure and outside shell. This is particularly beneficial for excellent charge and discharge capacity, and outstanding long-term cycling performance of the sulfur cathode as it significantly suppresses the shuttle effect and accommodates high volume change. As a consequence, we obtained a high initial capacity of 1142 mAh g^–1^ and an excellent stabilized capacity of 886 mAh g^–1^ at 0.2 C after 500 cycles: one of the best performances compared to those reported in the previous literature (Supplementary Table S1). We also observed unprecedented electrode integrity after 500 cycles and an absence of volume change after 200 cycles. Most importantly, we were able to produce hollow sandwiched sulfur nanospheres (HNPs) with PANI layers (S–PANI@S@S–PANI–HNPs) in large amounts using a very simple and sustainable preparation process. These superior properties suggest that the S–PANI@S@S–PANI–HNPs hybrid is a commercially viable and easily upscalable cathode material for large-scale high-performance Li–S batteries.

## RESULTS AND DISCUSSION

**Figure 1.**
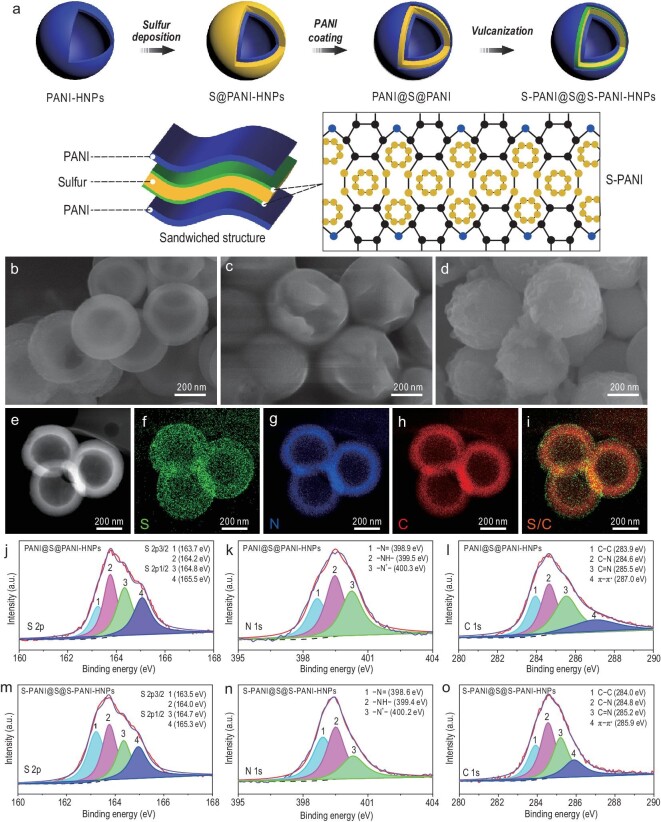
(a) Schematic diagram for the synthesis of sandwiched S–PANI@S@S–PANI–HNPs and the structure of the S–PANI backbone. SEM images of (b) PANI–HNPs, (c) S@PANI–HNPs and (d) S–PANI@S@S–PANI–HNPs. (e) High-angle annular dark-field scanning transmission electron microscopy (HAADF-STEM) image of S–PANI@S@S–PANI–HNPs and (f–i) the corresponding EDX mapping of S, N, C and S/C. High-resolution XPS spectra of (j and m) S 2p, (k and n) N 1s and (l and o) C 1s of PANI@S@PANI–HNPs and S–PANI@S@S–PANI–HNPs.

Figure 1a illustrates the steps of the upscalable synthesis process and structure of S–PANI@S@S–PANI–HNPs. First, PANI hollow nanoparticles (PANI–HNPs) were prepared through a very simple hydrothermal process [31]. The hierarchical hollow structure of PANI–HNPs possessed a smooth surface, a diameter of ∼400 nm and a shell thickness of ∼50 nm (Fig. 1b). Such a hollow structure can provide enough void space to accommodate the volume variation during the cycling process compared to solid sulfur nanoparticles [26]. In addition, the hollow structure avoids an inactive sulfur core in solid constructions, offering the possibility to make full use of active sulfur. Second, sulfur was deposited on the outer surface of PANI–HNPs to form S@PANI–HNPs. As shown in Fig. 1c, the S@PANI–HNPs retained the original spherical morphology of the PANI–HNPs with a rough surface and a diameter of ∼600 nm, indicating a sulfur layer thickness of ∼100 nm and very high S loading. Finally, a new PANI layer was coated on the S@PANI–HNPs, resulting in the PANI@S@PANI–HNPs structure with two PANI layers on both sides of the sulfur layer. This structure was then *insitu* vulcanized at 280°C to form the sandwiched S–PANI@S@S–PANI–HNPs architecture (Fig. 1d).

The diameter of the S–PANI@S@S–PANI–HNPs was measured to be ∼500 nm, with an outer PANI layer of thickness ∼50 nm. Importantly, the PANI layer on both sides of the sulfur layer not only facilitated high sulfur loading, optimal utilization of loaded sulfur and efficient electron transport, but also endowed their direct reaction with the sulfur layer to form a cross-linked, structurally stable S–PANI polymer network between S and PANI upon the *insitu* vulcanization. This provided a synergistically strong structural and chemical interaction with LiPSs from two sides of the sulfur layer, improving the electrochemical performance of the electrode material. A description of all the samples obtained at each step including reference samples and the final targeted sample can be found in Supplementary Table S2.

High-angle annular dark-field scanning transmission electron microscopy (HAADF-STEM) was performed to analyse the structure of the S–PANI@S@S–PANI–HNPs. Figure 1e shows a typical HAADF-STEM image, displaying the hollow structure of the monodispersed S–PANI@S@S–PANI–HNPs with a size of ∼500 nm, which is consistent with the scanning electron microscopy (SEM) observation (Fig. 1b–d). Figure 1f–i presents the corresponding energy dispersive spectroscopy (EDX) mapping images that show the elemental distribution of N, S and C. Sulfur was mainly detected in the middle layer (Fig. 1f), whereas N (Fig. 1g) and C (Fig. 1h) were homogeneously distributed in the inner and the outer shell. In addition, the X-ray diffraction (XRD) patterns showed that the S–PANI@S@S–PANI–HNPs, with the sandwiched structure, exhibit diffraction peaks of orthorhombic sulfur (JCPDS 08-0247), together with weak diffraction peaks of the emeraldine salt of PANI (Supplementary Fig. S1 and Supplementary Data) [13].

Fourier transform infrared spectroscopy (FTIR) and thermogravimetric analysis (TGA) were carried out to reveal the interaction between sulfur and PANI before and after *insitu* vulcanization. For comparison, measurement of the PANI@S@PANI–HNPs without *insitu* vulcanization was also performed. In the FTIR spectra shown in Supplementary Fig. S2, the C=C stretching vibration (1499 cm^−1^) assigned to benzenoid rings shifted to lower wave numbers, likely due to the substitution of H atoms on benzenoid rings by S atoms. The intensity of the C–H vibrational band at 1172 cm^−1^ was strongly reduced, further confirming the replacement of H atoms on aromatic rings by S atoms. It is known that there is no vibrational activity of sulfur from the range of 900–2000 cm^−1^ [32]. However, two new bands were noticed in the vicinity of 996 and 927 cm^−1^. These two peaks were likely caused by the vibrations of aromatic rings (Ar)–S and Ar–S–S–Ar, respectively [33,34]. These results indicate that after *insitu* vulcanization, sulfur reacts with PANI to form a cross-linked, structurally stable S–PANI backbone. This was further evidenced by mass loss and TGA curves (Supplementary Fig. S3). Apart from the endothermic peak of H_2_O desorption at ∼110°C, endothermic peaks at ∼275°C for PANI@S@PANI–HNPs and at ∼300°C for S–PANI@S@S–PANI–HNPs were observed, which correspond to sulfur loss [35]. The higher temperature for S loss suggests that sulfur in S–PANI@S@S–PANI–HNPs is thermally more stable than in PANI@S@PANI–HNPs, due to the formation of C–S and S–S bonds in the S–PANI@S@S–PANI–HNPs network. The further weight loss after ∼300°C for PANI@S@PANI–HNPs and after 320°C for S–PANI@S@PANI–HNPs is due to the carbonization of PANI [17,18]. From TGA analysis, the sulfur content of S–PANI@S@S–PANI–HNPs was determined to be ∼73%. Such a high sulfur loading is helpful to achieve a high energy density in Li–S batteries.

The chemical interaction between sulfur and PANI in PANI@S@PANI–HNPs and S–PANI@S@S–PANI–HNPs was further investigated using X-ray photoelectron spectroscopy (XPS) (Supplementary Fig. S4). The S 2p spectra of both samples were fitted with four peaks (Fig. 1j and m) [13]. Peaks 1 and 2 in PANI@S@PANI–HNPs at 163.7 and 164.2 eV correspond to the S 2p3/2 component peak of the phenyl ring linked S and the elemental S (S_8_), respectively, due to the electron-donating character of phenyl rings [36,37]. Compared to the PANI@S@PANI–HNPs, Peak 1 of S 2p3/2 in S–PANI@S@S–PANI–HNPs assigned to the phenyl ring shifted ∼0.2 eV to lower binding energy, suggesting electron migration from the phenyl rings to the S atom and strengthened C–S interaction after *insitu* vulcanization [32,38]. Besides, the peak area of S 2p3/2 belonging to the S linked to the phenyl ring significantly increased, indicating that more sulfur atoms were involved in strong interaction at 280°C. The peak area calculation indicated that ∼7% of the sulfur chemically interacted with polyaniline after *insitu* vulcanization at 280°C. Figure 1k and n presents high-resolution XPS spectra of N 1s of PANI@S@PANI–HNPs and S–PANI@S@S–PANI–HNPs. Three sub-peaks at 398.9, 399.5 and 400.3 eV of PANI@S@PANI–HNPs can be attributed to –N= , –NH– and –N^+^–, respectively [38]. After the *insitu* vulcanization, all these three peaks in S–PANI@S@S–PANI–HNPs shifted towards lower binding energies, indicating substitution of H atoms by sulfur. In particular, the ratio of [–N=]/[–NH–] in S–PANI@S@S–PANI–HNPs (0.97) was found to be higher than that in PANI@S@PANI–HNPs (0.67), further confirming the presence of chemically bonded sulfur in the S–PANI networks. Figure 1l and o shows the C 1s spectra that can be deconvoluted to the four peaks corresponding to C–C, C–N, C=N and π–π^*^ [39]. Compared to PANI@S@PANI–HNPs, C–C and C–N peaks in S–PANI@S@S–PANI–HNPs shifted to higher binding energies. This results from the incorporation of sulfur into the PANI network to form an S–PANI backbone, causing the polar character of the C–S bonds [13]. Additionally, the C 1s peak assigned to π–π^*^ shifted ∼1.1 eV to lower the binding energy after vulcanization, confirming the p–π conjugation formation endowing a more stable S–PANI network structure [38]. Further details are presented in Supplementary Tables S3–S5. The above results confirm that upon the *insitu* vulcanization process, part of the elemental sulfur reacts with PANI to form a cross-linked stereo S–PANI network with disulfide bonds between S and PANI (Fig. 1a). Meanwhile, the rest of the melted elemental sulfur diffused into the newly formed S–PANI network. This resulted in a dual physical (sandwiched effect) and C–S chemical bonding formation for effective confinement of sulfur species.

To prove the strong anchoring effect of the S–PANI network for sulfur species, ultraviolet visible (UV–Vis) spectroscopy was carried out; 0.02 g of bare sulfur nanoparticles (S–NPs), S@S–PANI–HNPs, PANI@S@PANI–HNPs and S–PANI@S@S–PANI–HNPs (Supplementary Table S2) were each mixed with 2 mL of 1 mM Li_2_S_6_/1,2-dimethoxyethane solution and each mixture was kept for 12 h (Supplementary Fig. S5). The UV spectra of the solutions show that the absorbance intensity of the band at ∼260 nm corresponding to the signal of the S_6_^2–^ species [40,41] decreases in the order Li_2_S_6_/S–NPs > Li_2_S_6_/S@S–PANI–HNPs > Li_2_S_6_/PANI@S@PANI–HNPs > Li_2_S_6_/S–PANI@S@S–PANI–HNPs and that of Li_2_S_6_/S–PANI@S@S–PANI–HNPs exhibits the weakest absorbance intensity of Li_2_S_6_ solution, indicating its lowest concentration of Li_2_S_6_ in the solution. Moreover, the optical photographs in the inset show that the Li_2_S_6_ solution with S–PANI@S@S–PANI–HNPs was nearly colorless after 12 h. Both UV–Vis spectroscopic studies and the optical photographs of Li_2_S_6_ with different samples in solution reveal the strongest anchoring capability of the S–PANI network toward the S_6_^2–^ species.

**Figure 2.**
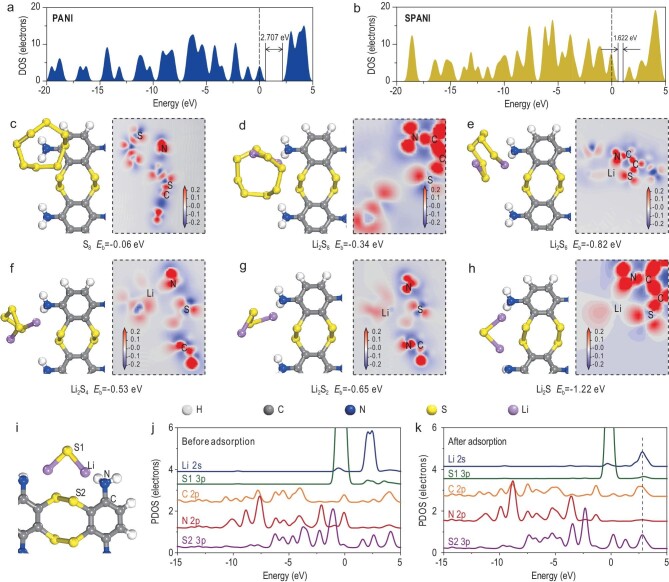
Density of state (DOS) spectra of (a) PANI and (b) S–PANI, where the zero points of the energy axis correspond to the Fermi level. The optimized configurations and corresponding electron density difference images (in the dashed frame) of (c) elemental S_8_, (d) Li_2_S_8_, (e) Li_2_S_6_, (f) Li_2_S_4_, (g) Li_2_S_2_ and (h) Li_2_S binding to S–PANI. Red and blue colors are used to represent the accumulation and depletion of charge, respectively. (i) PDOS spectrum of Li, S, N and C atoms (j) before and (k) after the Li_2_S binding to S–PANI. The zero points of the energy axis correspond to the Fermi level.

To further understand how the S–PANI backbone effectively anchors sulfur species by forming strong chemical bonds, density functional theory (DFT) calculations on pure PANI and S–PANI networks were performed. Figure 2a and b show their density of state (DOS) spectra. The bandgap of pure PANI (2.707 eV) is much larger than that of S–PANI (1.622 eV). This suggests that the S–PANI network formed through *insitu* vulcanization narrows the bandgap, facilitating electron transfer and the utilization of active materials.

The electronic adsorption energy (*E_b_*) of sulfur/LiPSs within the S–PANI host was then calculated using Equation 1 (Computational method). Generally, a more negative *E_b_* corresponds to a more stable adsorption system and a favored interconnection between sulfur species and the host [40,41]. The optimized configurations from sulfur to various LiPSs binding with S–PANI are presented in Fig. 2c–h. At the initial stage, the sulfur (S_8_) is adsorbed on S–PANI with a relatively weak binding energy of −0.06 eV (Fig. 2c). As the lithiation proceeds, Li_2_S_8_, Li_2_S_6_, Li_2_S_4_, Li_2_S_2_ and Li_2_S exhibit binding energies of −0.34, −0.82, −0.53, −0.65 and −1.22 eV respectively, which is much higher than conventional carbon material with −0.34 eV [8,40]. The electron density difference images (dashed areas in Fig. 2c–h) further highlight the charge rearrangement at the Li, C, N and S atoms after Li_x_S_y_ adsorption. There is an obvious electron density accumulation in the N and S regions, suggesting a strong interaction between the Li of LiPSs and the C, N and S of S–PANI. These results are also confirmed by the partial density of state (PDOS) study (Fig. 2i–k). The orbitals of Li 2s of LiPSs and C 3p, N 2p and S 3p of S–PANI overlapped at ∼2.6 eV, exhibiting beneficial electron migration among LiPSs and S–PANI [42]. An intrinsic atomic orbital hybridization between Li 2s of LiPSs and C 2p, N 2p and S 3p of S–PANI favors a strong chemical bonding between LiPSs and S–PANI, thereby suppressing the separation of LiPSs from the S–PANI host and facilitating superior stability for long-term discharge/charge cycling. Compared to the weak physical confinement of LiPSs in carbon matrix with low binding energy (0.34 eV), which leads to the detachment and dissolution of active materials [8,40], our strategy reveals a new type of chemical confinement via orbital hybridization between Li 2s of LiPSs and S 3p, C 2p and N 2p of S–PANI host, offering much stronger chemical anchoring by the S–PANI network for sulfur species [36]. Furthermore, it is shown that the preparation of hollow sandwiched sulfur nanospheres (HNPs) with double-polyaniline (PANI) layers (S–PANI@S@S–PANI–HNPs) is very simple and can be easily upscaled for production in large quantities.

**Figure 3.**
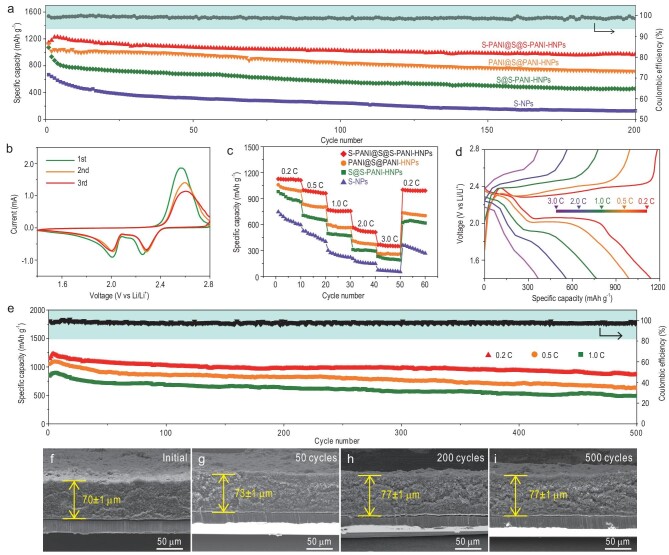
(a) Cycling performance of S–NPs, S@PANI–HNPs, PANI@S@PANI–HNPs and S–PANI@S@S–PANI–HNPs at 0.2 C. (b) CV curves of S–PANI@S@S–PANI–HNPs at a sweep rate of 0.2 mV s^–1^ in the voltage range of 1.4–2.8 V vs Li/Li^+^. (c) Rate performance of S–NPs, S@PANI–HNPs and S–PANI@S@S–PANI–HNPs. (d) Charge/discharge profiles of S–PANI@S@S–PANI–HNPs at various current densities. (e) Cycling performance of S–PANI@S@S–PANI–HNPs at 0.2, 0.5 and 1.0 C for 500 cycles. (f–i) Side-view SEM images of the S–PANI@S@S–PANI–HNPs electrode at selected cycles at 0.2 C.

The structurally and chemically dual-anchored LiPSs in such a sandwiched hollow sphere are next evaluated for their electrochemical performance. For comparison, S–NPs, S@S–PANI–HNPs and PANI@S@PANI–HNPs are also tested. Figure 3a presents their cycling performance at 0.2 C. S–NP electrodes exhibited a rapid decay in specific capacity with a short cyclic life. The initial capacity was only 668 mAh g^–1^ and dropped to 130 mAh g^–1^ after 200 cycles with a large capacity decay of 0.4% per cycle. On the one hand, the sulfur in S–NPs was directly exposed to the electrolyte, producing soluble LiPSs, leading to an intense shuttle effect and severe loss of active sulfur. On the other hand, the large volumetric expansion of the solid S–NPs during the discharge/charge process can pulverize the cathode structure, resulting in low coulombic efficiency and a short lifespan. Compared to S–NPs, S@S–PANI–HNPs exhibited an improved initial discharge capacity of 985 mAh g^–1^ and capacity retention of 670 mAh g^–1^ after 200 cycles. Although the improvement reveals the advantage of the chemical confinement for LiPSs by a single S–PANI network, the outer sulfur layer still remains in direct contact with the electrolyte, resulting in the dissolution of LiPSs. Meanwhile, PANI@S@PANI–HNPs displayed a further improved specific capacity of 1082 mAh g^–1^ and capacity retention of 770 mAh g^–1^ after 200 cycles, demonstrating the effectiveness of the structural confinement provided by the double PANI layer, with a capacity decay of 0.14% per cycle. Notably, the hollow structures and the structural confinement can significantly contribute to the improvement of electrochemical performance. For the S–PANI@S@S–PANI–HNPs electrode, the cycle life was greatly improved because of the effective dual chemical confinement of sulfur species within the double S–PANI network via a strong atomic orbital hybridization of Li 2s of LiPSs and S 3p, C 2p and N 2p of S–PANI in addition to the structural confinement (Fig. 1a). The initial discharge capacity of S–PANI@S@S–PANI–HNPs was observed to be 1142 mAh g^–1^, followed by a slight activation process due to the tardive electrolyte infiltration into the well-capsulated sandwiched structure after vulcanization. The capacity was retained at 996 mAh g^–1^ after 200 cycles, with a significantly reduced capacity decay of only ∼0.06% per cycle, showing excellent stability of the electrode. As shown in Supplementary Fig. S6, the transmission electron microscopy (TEM) image and the corresponding EDX mappings confirm that the morphology was well preserved after 100 cycles. The sulfur was well confined within the sandwiched structure, which further confirms the efficient protection via the atomic orbital hybridization of S–PANI@S@S–PANI–HNPs on structure stability. Figure 3b shows the CV curves of the S–PANI@S@S–PANI–HNPs electrode for the first cycles. The two main reduction peaks at ∼2.3 and ∼2.0 V can be assigned to the multistep reduction of elemental sulfur during the cathodic scan process. The peak at ∼2.3 V corresponds to the reduction of sulfur to high-order LiPSs (Li_2_S*_n_*, 4 ≤ *n* ≤ 8) and the second peak at ∼2.0 V represents a further reduction of the high-order LiPSs to Li_2_S_2_ and Li_2_S. In the subsequent anodic scans, an oxidation peak was observed at ∼2.5 V, associated with the oxidation of the low-order LiPSs to high-order LiPSs [43]. The typical peaks in the CV plot indicate that the solvent molecules of the electrolyte have good contact with sulfur and that the redox reaction occurs. The generated soluble polysulfide (Li_2_S*_n_*, 4 ≤ *n* ≤ 8) is firmly encapsulated within the cathode by our sandwich structure, thus eliminating the excessive shuttle effect. Significantly, no obvious changes in the CV peak positions or peak current can be observed in the following scans, confirming the exceptional electrochemical stability of S–PANI@S@S–PANI–HNPs. The initial three discharge/charge profiles of the S–PANI@S@S–PANI–HNPs electrode with a cut-off voltage window of 1.4–2.8 V at the current density of 0.2 C are presented in Supplementary Fig. S7. The two plateaus observed at ∼2.3 and ∼2.0 V versus Li/Li^+^ are associated with the two-step reduction during the discharge process, consistently with the CV results. The initial discharge capacity of the S–PANI@S@S–PANI–HNPs electrode reached a high value of 1142 mAh g^–1^, indicating very high sulfur utilization. It is worth noting that after three cycles, the discharge capacity gradually increased to 1228 mAh g^–1^, strongly suggesting an increased utilization of sulfur.

The rate performance of S–PANI@S@S–PANI–HNPs (Fig. 3c) indicates very stable capacities of 1157, 998, 770, 565 and 366 mAh g^–1^ at a current density of 0.2, 0.5, 1.0, 2.0 and 3.0 C, respectively. When the current density dropped to 0.2 C, a reversible capacity of 1002 mAh g^–1^ was obtained, suggesting excellent stability and reversibility at different rates. Moreover, the typical plateaus in the discharge curves (Fig. 3d) were well maintained during cycling even at a high current density of 3.0 C. This indicates that the double S–PANI layer network significantly improves the kinetic behavior of the cathode.

Excellent long-cycle performance of S–PANI@S@S–PANI–HNPs electrode at current densities of 0.2, 0.5 and 1.0 C for 500 cycles highlights the advantages of the unique sandwiched hollow structure with dual structural and chemical confinement of sulfur species on either side of the sulfur layer. After an initial discharge capacity of 1142 mAh g^–1^, a discharge capacity of 886 mAh g^–1^ at 0.2 C after 500 cycles was achieved (Fig. 3e). This corresponds to a very-low-capacity decay of ∼0.04% per cycle. In addition, the coulombic efficiency always remained at >98%, indicating high reversibility even for long-term cycles. Furthermore, the initial discharge capacities of 1053 and 845 mAh g^–1^ at 0.5 and 1.0 C, respectively, were achieved, with a low decay of 0.08% per cycle after 500 cycles. Supplementary Figures S8–S10 show the corresponding discharge/charge profiles at 0.2, 0.5 and 1.0 C, which maintain the typical two-plateau behavior even after 500 cycles, revealing excellent kinetics and stability at high current density.

As shown in Supplementary Fig. S11, before cycling, the S–PANI@S@S–PANI–HNPs electrode exhibited a much lower charge-transfer resistance than the S–NPs electrode, indicating a greatly enhanced electrical conductivity by the PANI. The beneficial gradual decrease of the total impedance in the S–PANI@S@S–PANI–HNPs electrode after cycling can be attributed to the reduction of solid sulfur to soluble LiPSs, which are redistributed in the S–PANI network during the discharge/charge process. This redistribution of LiPSs within the cathode region strongly promotes the charge-transfer process between the PANI and the active sulfur species [44]. The DOS spectrum of S–PANI@S@S–PANI–HNPs (Supplementary Fig. S12) revealed a much narrower bandgap (1.176 eV) than that of sulfur (3.336 eV). It confirms more efficient utilization of active materials by the S–PANI network, consistently with the electrochemical impedance spectroscopy (EIS) results.

The disassembled S–NPs and S–PANI@S@S–PANI–HNPs electrodes before and after cycling were analysed using SEM. Compared to the initial spherical structure of S–NPs (Supplementary Fig. S13a), a significant number of broken spheres and agglomerated insulating Li_2_S/Li_2_S_2_ nanoparticles on the surface of the S–NPs electrode were observed after cycling (Supplementary Fig. S13b and c). On the other hand, the S–PANI@S@S–PANI–HNPs electrode retained its original morphology after 500 cycles (Supplementary Fig. S14a–c). The sandwiched S–PANI@S@S–PANI–HNPs with dual structural and chemical confinement of active sulfur species maintained total electrode integrity for high-performance Li–S batteries, as schematically illustrated in Supplementary Figs S13d and S14d. The thickness of the S–PANI@S@S–PANI–HNPs electrodes before and after cycling was carefully investigated to show the volume change (Fig. 3f–i). The side-view SEM images revealed an initial thickness of 70 ± 1 μm and a thickness of 73 ± 1 μm after 50 cycles at 0.2 C, reaching a stable thickness of 77 ± 1 μm after 200 cycles. No change in the thickness was noted from 200 to 500 cycles, showing the excellent textural stability of the electrode. The maximum change in thickness corresponds to a very low value of ∼10%. By contrast, the thickness of the S–NPs electrode increased from 64 mm at the beginning of cycling to 70 mm after 50 cycles and further to 80 mm after 200 cycles with a continuous volume expansion of ∼25% (Supplementary Fig. S15). This confirms that the sandwiched hollow nanostructure can efficiently accommodate sulfur volume variation to ensure electrode integrity, leading to high-performance Li–S batteries.

**Figure 4.**
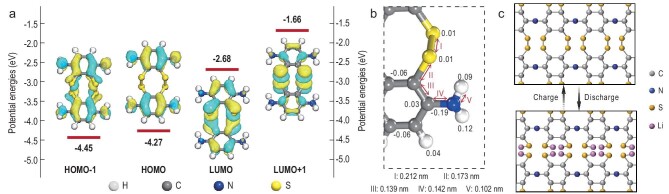
(a) Frontier molecular orbitals, (b) Hirshfeld charge and bond length analysis of (I) S–S, (II) C–S, (III) C–C, (IV) C–N and (V) N–H of S–PANI. (c) Schematic illustration of the S–PANI network during the discharge/charge process.

As mentioned above, the S–PANI network formed by *insitu* vulcanization plays an important role in high reversible and stable electrochemical performance. The frontier molecular orbitals from the HOMO–1 (Highest Occupied Molecular Orbital) to the LUMO+1 (Lowest Unoccupied Molecular Orbital), Hirshfeld charge and the bond length of the S–PANI network were further analysed to identify the reaction of the chemically linked S in the S–PANI network during the discharge/charge process. Generally, the HOMO refers to the ground-state potential of the oxidizing reaction, while the LUMO refers to the excitation-state potential that mainly corresponds to an electron upgrade to this orbital [45]. It can be seen that the HOMO–1/HOMO is well localized in the aromatic benzene ring donor while the LUMO/LUMO+1 is mainly contributed by the S–S linkage (Fig. 4a). This indicates that the S–S bond is the most active region in S–PANI to accept electrons, due to the gradually increased electron density in the electronic transition process from HOMO–1 to LUMO+1. It is consistent with the Hirshfeld charge analysis shown in Fig. 4b, which is widely used to predict the possible reaction site for electron transfer in chemical processes [46].

The S–S bond (I: 0.212 nm) exhibits the longest bond length compared to the others in S–PANI, such as C–S (II: 0.173 nm) and C–N (III: 0.139 nm), indicating that the chemical S–S bond tends to readily expose S to participate in the delithiation/lithiation process. As a consequence, the S–S bonds exhibit electrochemical activity from the intramolecular electro-catalytic effect of the aniline moiety, as well as the improved recombination efficiency from the confined polymer structure that interconnects with the S–S bonds (Fig. 4c) [38]. Therefore, during the discharge process, the side chains of the S–S functional groups firstly accept electrons, then are depolymerized and combined with lithium ions, while the main chain of S–PANI is stable. In the charging process, the S–S bonds recombine. The release of the chemically linked S in the S–PANI networks can explain the gradual increase in discharge capacity in the first several cycles. This suggests that the atomic orbital hybridization between Li 2s of LiPSs and S 3p, C 2p and N 2p of S–PANI is highly reversible. With the reversible depolymerization and recombination of the chemically linked S in S–PANI, a stable capacity is achieved even at a high current rate of 1.0 C after 500 cycles.

## CONCLUSION

We have demonstrated a new and commercially viable strategy via a new concept—an atomic orbital hybridization of Li 2s of LiPSs and S 3p, C 2p and N 2p—to synthesize hierarchical hollow sandwiched sulfur nanospheres with a double S–PANI network, possessing structural and dual chemical confinement of sulfur species, and volume accommodation. Such a functional cathode with a facile and immediately upscalable synthesis procedure exhibits excellent electrochemical performance with a high initial capacity of 1142 mAh g^–1^ and long-term cyclic stability of 886 mAh g^–1^ after 500 cycles at 0.2 C and a total electrode integrity. Even at a high current of 1.0 C, the electrode demonstrates a very-low-capacity decay of 0.08% per cycle. Furthermore, DFT calculations confirm that the cross-linked and structurally stable sandwiched S–PANI@S@S–PANI–HNPs cathode efficiently confines LiPSs via an orbital hybridization of Li 2s of LiPSs and S 3p, C 2p and N 2p of S–PANI in addition to the sandwich structural effect and strongly suppresses the volume change. Our work presents a commercially viable strategy and offers important insights to suppress the shuttle effect and volume expansion, and to improve electrochemical reaction kinetics and stability, thus taking the practical implementation of Li–S batteries a step closer.

## MATERIALS AND METHODS

### Bare sulfur nanoparticles (S–NPs)

The S–NPs were synthesized by adding 0.8 mL of HCl (10 M) to 100 mL of Na_2_S_2_O_3_·5H_2_O solution (0.04 M) containing a low concentration of polyvinylpyrrolidone (PVP, Mw ∼ 55 000). After stirring for 2 h, the S–NPs were washed by centrifugation and dried in a vacuum at 60°C for 6 h.

### PANI hollow nanoparticles (PANI–HNPs)

First, 0.5 g of aniline monomer and 5.5 mL of H_3_PO_4_ were dissolved into 200 mL of deionized water with vigorous stirring for 5 min to form a uniform solution. Then, 1.135 g of H_2_O_2_ and 16 mg of FeCl_3_ were subsequently added to the above solution. After being fully stirred, the mixture was transferred into a Teflon-lined stainless-steel autoclave and kept at 140°C for 6 h in a digital temperature-controlled oven. Then the autoclave was naturally cooled to room temperature. The precipitate was filtered and washed using deionized water/ethanol until the filtrate became colorless, followed by drying in a vacuum at 60°C for 8 h.

### S@S–PANI–HNPs

First, 0.03 g of PANI–HNPs, 1.6 g of PVP and 3 g of Na_2_S_2_O_3_·5H_2_O were dissolved in 160 mL of deionized water with sufficient stirring and ultrasound. Next, 25 mL of HCl (1.5 M) was added to the mixture dropwise. After being stirred for 2 h, the precipitates were collected by filtration and washed several times using deionized water/ethanol and dried at 60°C for 12 h. S@S–PANI–HNPs were obtained after vulcanization in an argon atmosphere at 280°C for 10 h.

### S–PANI@S@ S–PANI–HNPs

First, 0.15 g of S@PANI hollow nanoparticles were dispersed in 100 mL of deionized water by ultrasound treatment. Later, 120 μL of aniline monomer, 240 μL of HCl and 0.2 g of (NH_4_)_2_S_2_O_8_ were added into the above suspension and stirred at room temperature until the color changed to dark green. The precipitates were then collected after filtration and washed several times using deionized water/ethanol. The S–PANI@S@S–PANI–HNPs were obtained through a subsequent vulcanization process under an argon atmosphere at 280°C for 10 h. For comparison, PANI@S@PANI–HNPs without the vulcanization process was also prepared. Description of all the studied samples can be found in Supplementary Table S2.

### Computational method

All DFT calculations were carried out using the Dmol^3^ and CASTEP package of Material Studio, with the generalized gradient approximation and Perdew-Burkee Ernzerhof functions. The HOMO/LUMO atomic orbital and the frontier orbital energies were performed using Dmol^3^. The DOS/PDOS and electron density differences were performed using CASTEP. An S–PANI molecule consists of 12 C, 12 H, 4 N and 4 S. The maximum force of 0.05 eV Å^–1^ was set to optimize the structural calculation accuracy. The energy convergence was set to 2.0 × 10^–6^ eV atom^–1^. The electronic adsorption energy (*E_b_*) was defined as:

(1) \begin{equation*} {{\boldsymbol{E}}_{\boldsymbol{b}}} = {{\boldsymbol{E}}_{{\boldsymbol{L}}{{\boldsymbol{i}}_2}{{\boldsymbol{S}}_{\boldsymbol{x}}}/{\boldsymbol{host}}}}{\boldsymbol{\ }} - {{\boldsymbol{E}}_{{\boldsymbol{L}}{{\boldsymbol{i}}_2}{{\boldsymbol{S}}_{\boldsymbol{x}}}}} - {{\boldsymbol{E}}_{{\boldsymbol{host}}}}, \end{equation*}

where ${{\boldsymbol{E}}_{{\boldsymbol{L}}{{\boldsymbol{i}}_2}{{\boldsymbol{S}}_{\boldsymbol{x}}}/{\boldsymbol{host}}}}{\boldsymbol{\ }}$is the electronic energy of the S–PANI and Li_2_S*_x_* (Li_2_S*_x_*, 1 ≤ *x* ≤ 8) complex, and ${{\boldsymbol{E}}_{{\boldsymbol{L}}{{\boldsymbol{i}}_2}{{\boldsymbol{S}}_{\boldsymbol{x}}}}}$ and ${{\boldsymbol{E}}_{{\boldsymbol{host}}}}$ stand for the electronic energy of the standalone S–PANI and Li_2_S*_x_* molecules, respectively.

## Supplementary Material

nwac078_Supplemental_File
