# Supplementary material for: Unprecedented strong and reversible atomic orbital hybridization enables a highly stable Li–S battery
Source: Natl Sci Rev. 2022 Apr 21;9(7):nwac078. doi: 10.1093/nsr/nwac078 (PMC9273299; doi:10.1093/nsr/nwac078)
Supplement: nwac078_Supplemental_File [file nwac078_supplemental_file.docx]

Supporting Information

**Unprecedented Strong and Reversible Atomic Orbital Hybridization Enables Highly Stable Li-S Battery**

Min Yan,^1,2^ Wenda Dong,^1^ Fu Liu,^3^ Lihua Chen,^1^ Tawfique Hasan,^4^ Yu Li,^1*^ and Bao-Lian Su^1, 5,*^

*^1^* *State Key Laboratory of Advanced Technology for Materials Synthesis and Processing, Wuhan University of Technology, Wuhan 430070, China; Email: yu.li@whut.edu.cn; baoliansu@whut.edu.cn*

*^2^* *Hubei Key Laboratory of Plasma Chemistry and Advanced Materials, Hubei Engineering Technology Research Center of Optoelectronic and New Energy Materials, Wuhan Institute of Technology, Wuhan 430205, China.*

*^3^* *School of Materials Science and Engineering, Zhejiang University, Hangzhou 310027, China.*

*^4^* *Cambridge Graphene Centre, University of Cambridge, Cambridge CB3 0FA, UK.*

*^5^* *Laboratory of Inorganic Materials Chemistry (CMI), University of Namur, Namur B-5000, Belgium; Email: bao-lian.su@unamur.be.*

**MATERIALS AND METHODS**

All the chemicals were purchased from Sigma-Aldrich and used as received without further purification.

Materials Characterization

X-ray diffraction patterns were obtained using a Bruker diffractometer (D8 advance) at 40 kV, 40 mA, with Cu Kα radiation (λ=1.54056 Å). The thermogravimetric analysis was recorded using a thermal analyzer (Setaram, Labsys Evo) in a nitrogen atmosphere with a temperature ramp of 1 °C min^-1^. The morphology of all the products was analysed using images from Hitachi S4800 scanning electron microscopy equipped with a field-emission gun at an accelerating voltage of 5 kV. High angle annular dark field-scanning transmission electron microscopy (HAADF-STEM) and energy dispersive spectroscopy (EDX) were performed using FEI Tecnai Osiris electron microscope fitted with a Super-X windowless EDX detector, operated at 200 kV. Fourier transform infrared (FT-IR) spectra were recorded on a Bruker Vertex 80 V FT-IR spectrometer using the KBr pellet technique in the range of 500-2000 cm^-1^. X-ray photoelectron spectroscopy (XPS) analysis ws done on a Thermo Fisher ESCALAB 205Xi. Deconvolution of the XPS spectra was performed with the Casa XPS program with Gaussian-Lorentzian functions.

Electrochemical measurement

The working electrode was prepared by mixing 75 wt% active material, 15 wt% acetylene black and 10 wt% polyvinylidene fluoride binder in N-methyl pyrrolidinone. The slurry was homogeneously coated on an aluminium foil current collector. The electrode was dried at 60 °C for 12 h under vacuum and subsequently cut into disks with a diameter of 12 mm. On average, the areal mass loading of sulfur was ~2.5 mg cm^-2^. Note that the specific capacities were based on the mass of sulfur only. The electrochemical measurements were performed using CR2025 coin cells and lithium metal as the counter electrodes. The electrolyte was 1.0 M lithium bis(trifluoromethanesulfonyl)imide in 1,3-dioxolane and 1,2-dimethoxyethane (volume ratio, 1:1) with 1 wt% LiNO_3_ as an additive. The volume of electrolyte injected in coin cells was controlled at ~10 µL/mg of sulfur. The coin cells were assembled in a glove box under an argon atmosphere. The galvanostatic charge/discharge tests were carried out on a LAND CT2001A battery tester in a potential range of 1.4-2.8 V (versus Li/Li^+^). Cyclic voltammetry was studied using a CHI 660D electrochemical workstation, with a scan rate of 0.2 mV/s. Electrochemical impedance spectra were measured with an electrochemical workstation (Autolab PGSTAT 302N) in the frequency range from 100 kHz to 10 MHz.

**
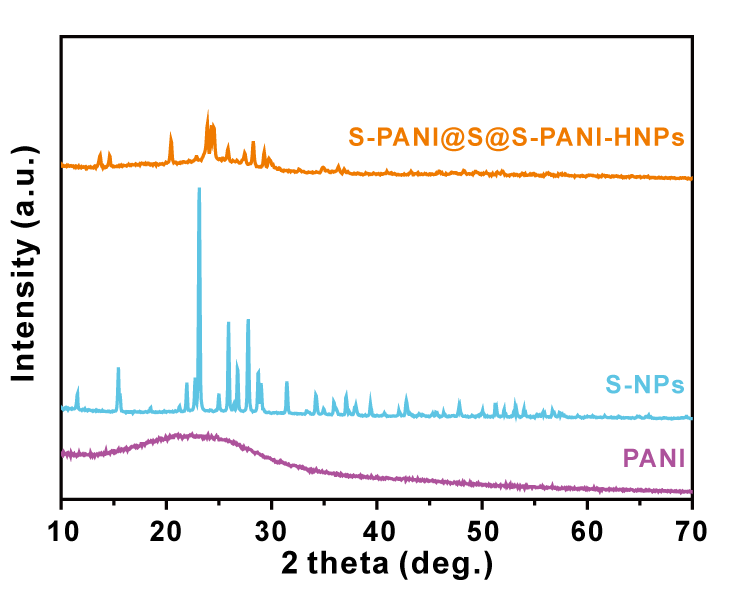
**

**Figure S1.** XRD patterns of PANI, S-NPs and S-PANI@S@S-PANI-HNPs.


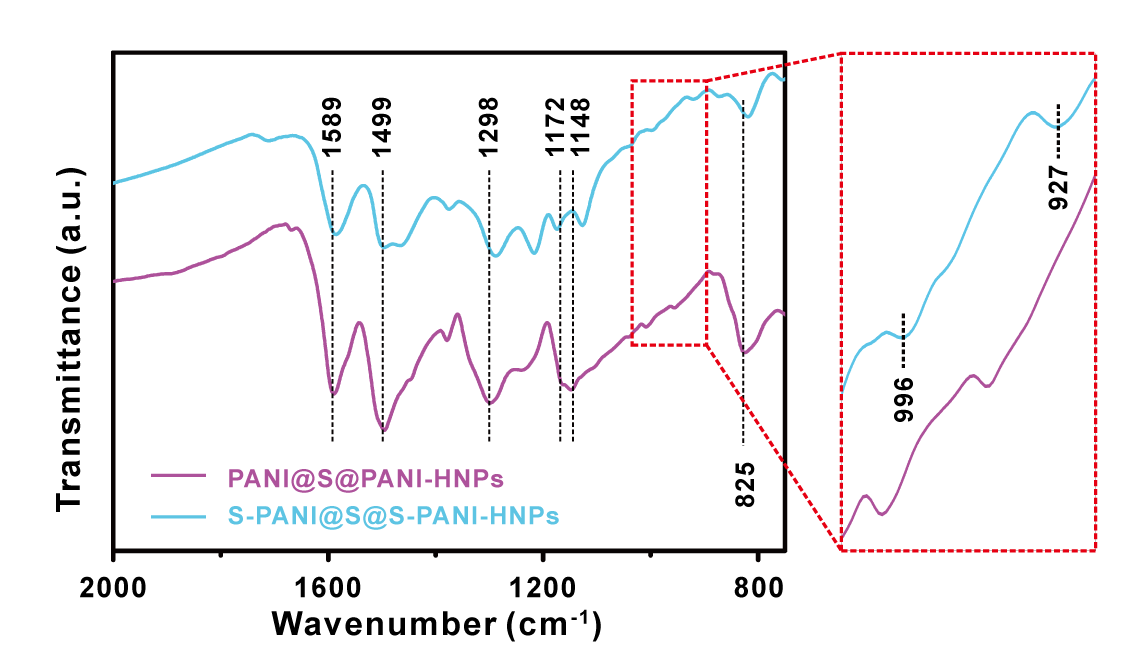


**Figure S2.** FT-IR spectra of PANI@S@PANI-HNPs and S-PANI@S@S-PANI-HNPs, the enlarged spectra are in the dotted box. The characteristic peaks of PANI are located in the 1000-2000 cm^-1^ region, 1589, 1499, 1298 and 1148 (825) cm^-1^ peaks are attributed to the C=N, C=C, C–N and in-plane C–H (out-of-plane C–H) stretching vibrations, respectively.

**
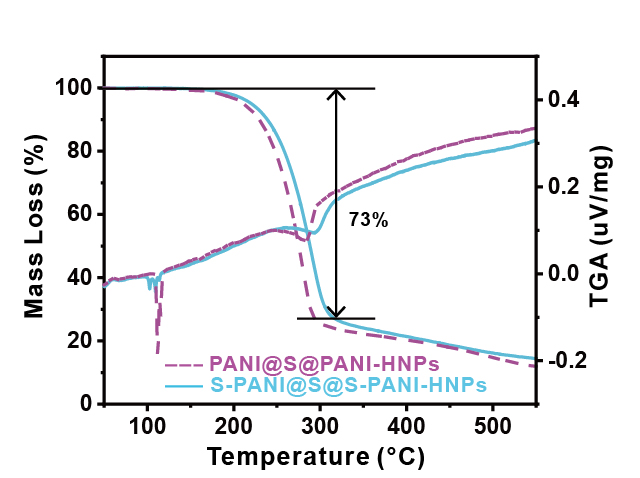
**

**Figure S3.** Mass loss and TGA curves of PANI@S@PANI-HNPs and S-PANI@S@S-PANI-HNPs.


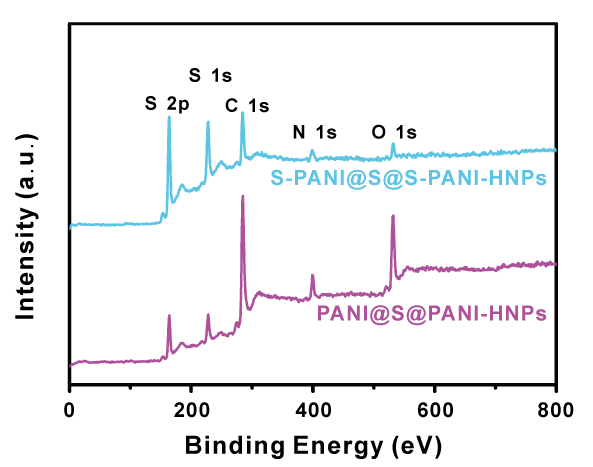


**Figure S4.** XPS spectra of PANI@S@PANI-HNPs and S-PANI@S@S-PANI-HNPs.


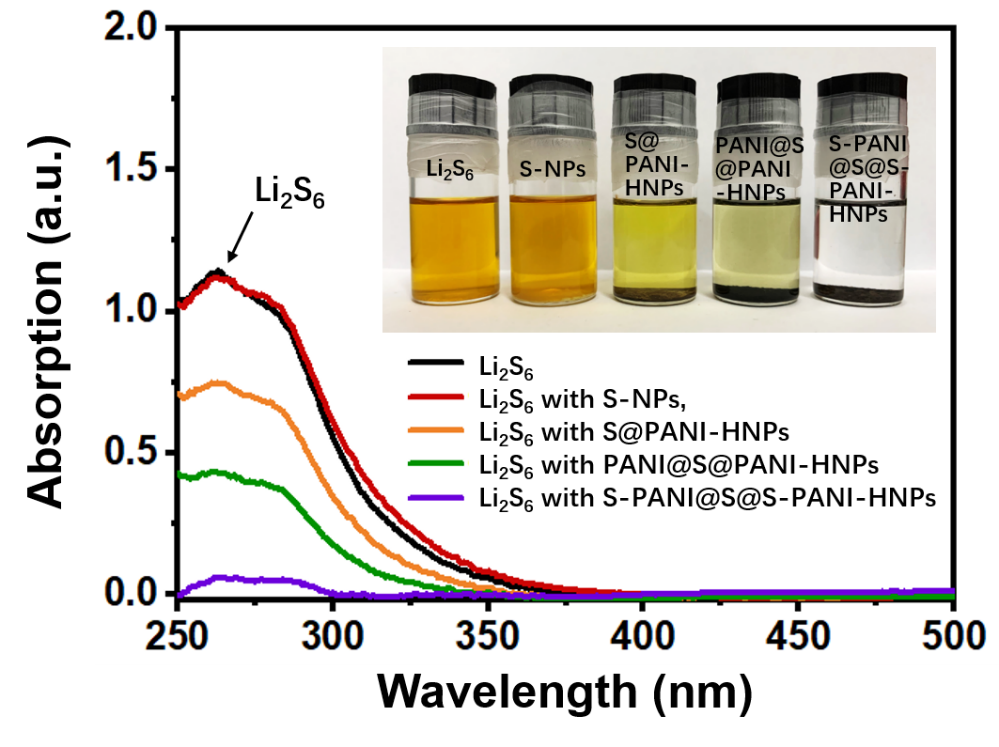


**Figure S5.** UV–Vis spectroscopy of the Li_2_S_6_ adsorption on S-NPs, S@S-PANI-HNPs, PANI@S@PANI-HNPs and S-PANI@S@S-PANI-HNPs, with the corresponding optical photographs.


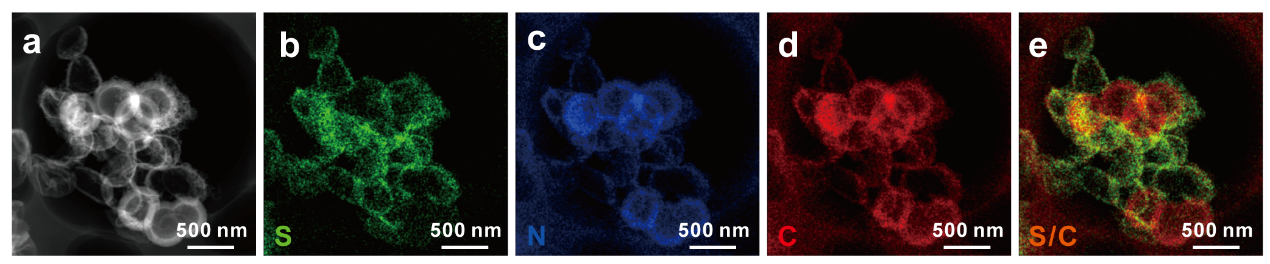


**Figure S6.** (a) The HAADF-STEM image and (b-e) corresponding EDX mappings of S, N, C and S/C of S-PANI@S@S-PANI-HNPs after 100 cycles.


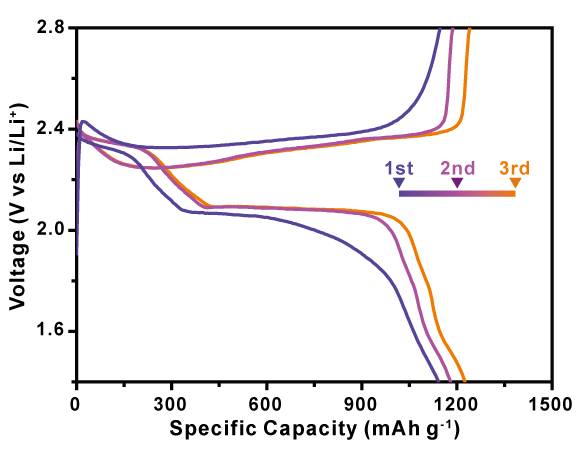


**Figure S7.** Initial three discharge/charge curves of the S-PANI@S@S-PANI-HNPs electrode at 0.2 C.


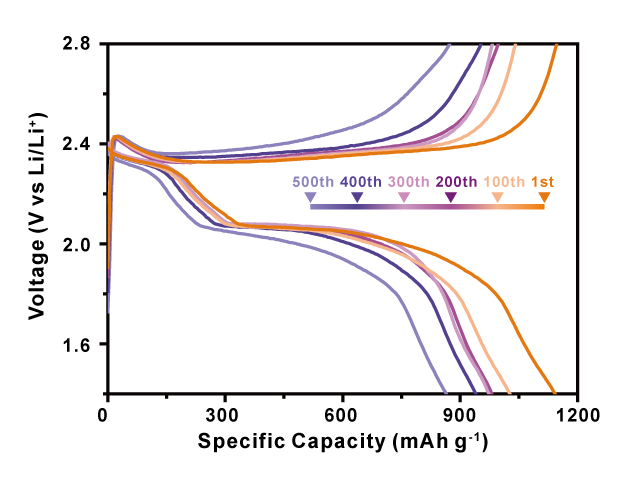


**Figure S8.** Discharge/charge curves of the S-PANI@S@S-PANI-HNPs electrode in different cycles at 0.2 C.


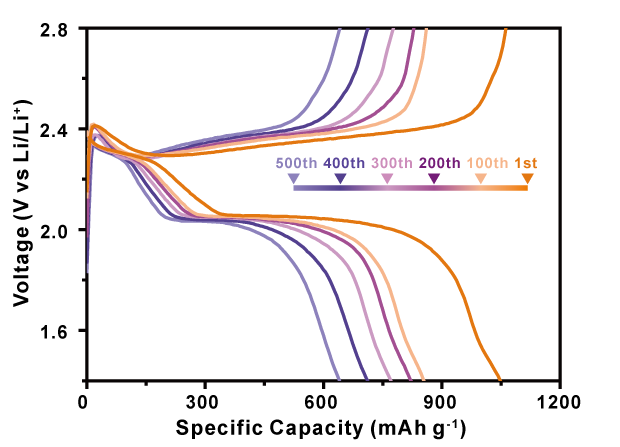


**Figure S9.** Discharge/charge curves of the S-PANI@S@S-PANI-HNPs electrode in different cycles at 0.5 C.


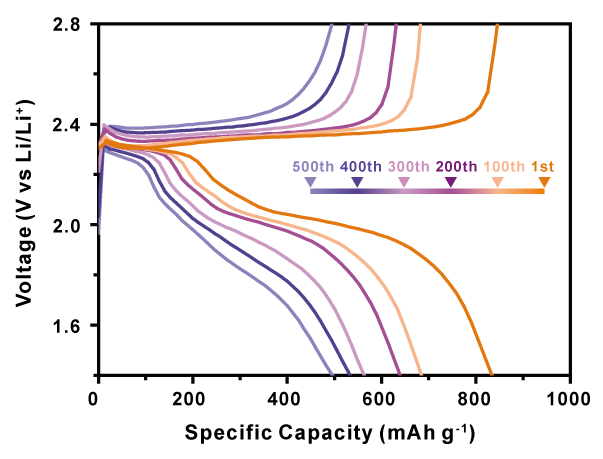


**Figure S10.** Discharge/charge curves of the S-PANI@S@S-PANI-HNPs electrode in different cycles at 1.0 C.


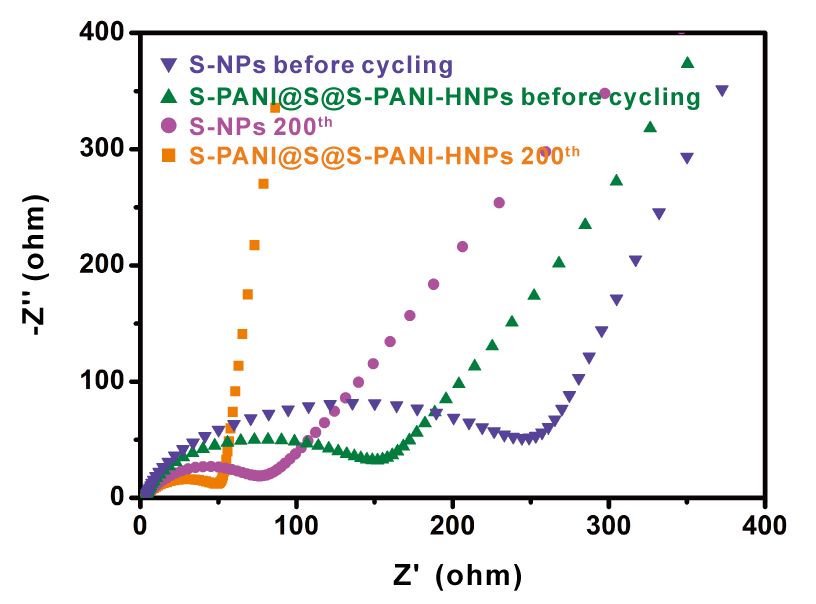


**Figure S11.** EIS spectra of S-NPs and S-PANI@S@S-PANI-HNPs before and after cycling.


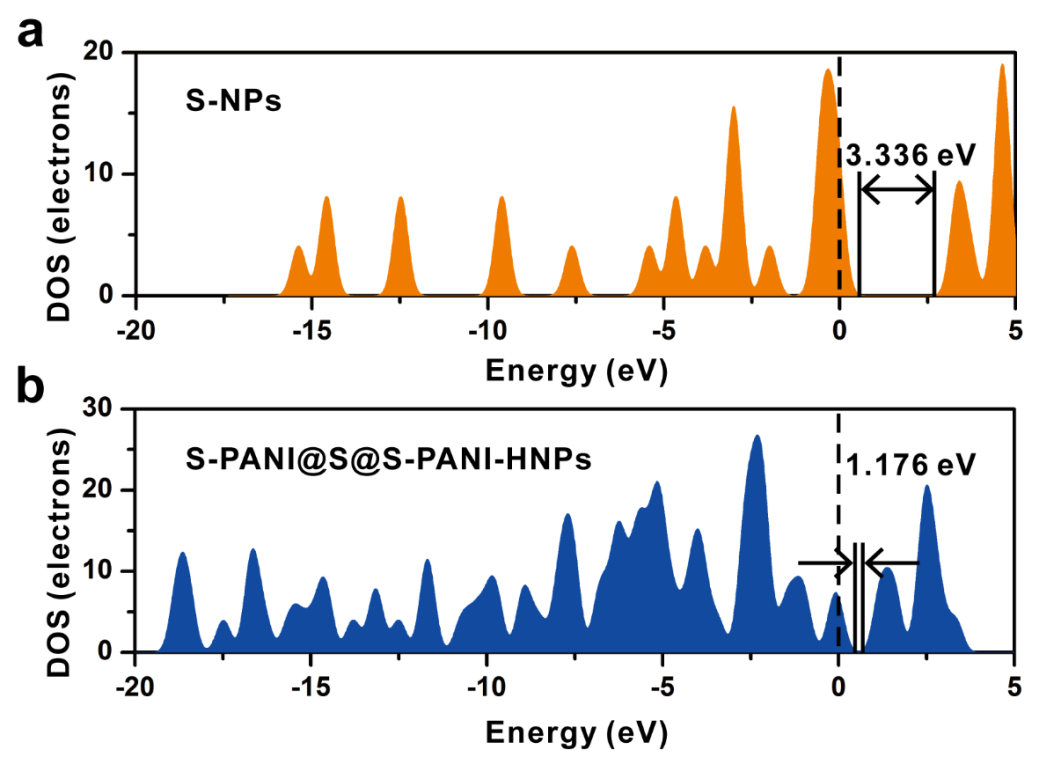


**Figure S12**. DOS spectrum of (a) S-NPs and (b) S-PANI@S@S-PANI-HNPs. The zero point of the energy axis corresponds to the Fermi level.


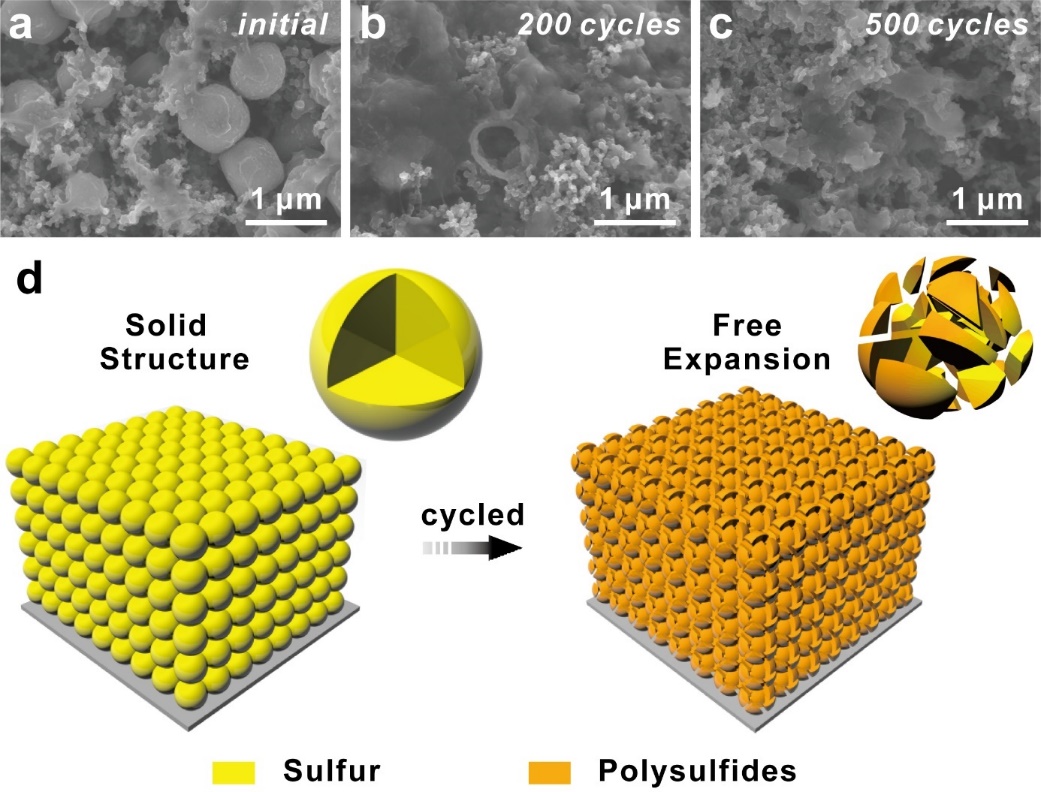


**Figure S13.** SEM images of the disassembled S-NPs electrode (a) before and (b) after 500 cycles at 0.2 C, and (c) schematic illustration of the S-NPs electrode for cycling.


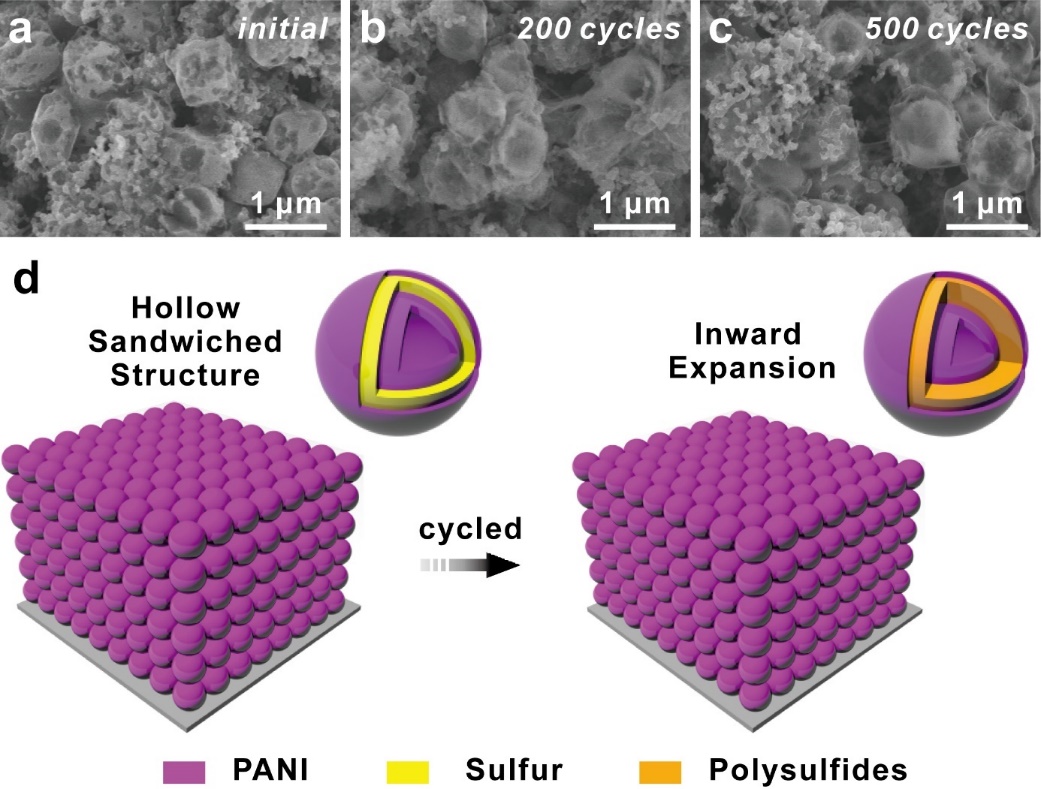


**Figure S14.** SEM images of the disassembled S-PANI@S@S-PANI-HNPs electrode (a) before and (b) after 500 cycles at 0.2 C, and (c) schematic illustration of the S-PANI@S@S-PANI-HNPs electrode for cycling.


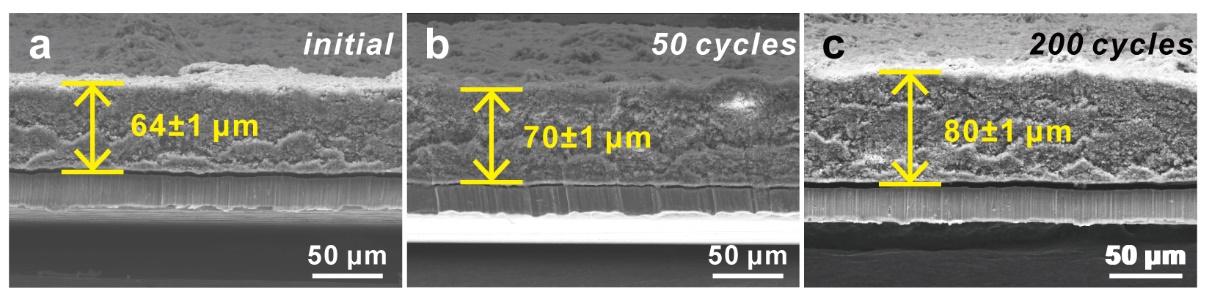


**Figure S15.** Side-view SEM images of the S-NPs electrode at selected cycles at 0.2 C.

**Table S1** Comparison of the electrochemical performances with the best state-of-the-art references.

| Composite | Cycling rate | Cycle number | Capacity  /mAh g^-1^ | Capacity decay rate  /% | Sulfur content  /% | Sulfur loading  /mg cm^-2^ | Ref. |
| --- | --- | --- | --- | --- | --- | --- | --- |
| Yolk-shell S-PANI | 0.2 C | 200 | 765 | 0.15 | 58 | 2 | [1] |
|  | 0.5 C | 200 | 628 | 0.16 |  |  |  |
| g-C_3_N_4_/S75 | 0.2 C | 500 | 620 | 0.06 | 75 | 1.5 | [2] |
| TiO@C hollow spheres | 0.2 C | 500 | 750 | 0.07 | 70 | 1.5 | [3] |
|  | 0.5 C | 500 | 630 | 0.08 |  |  |  |
| PPY-MnO_2_ nanotubes | 0.2 C | 200 | 985 | 0.15 | 70 | 1-2 | [4] |
| Ni/Fe layered double hydroxide | 0.2 C | 200 | 725 | 0.17 | 70 | 2-3 | [5] |
| DLHC/S@PEDOT:PSS | 0.2 C | 200 | 590 | 0.23 | 55 | 1 | [6] |
|  | 1 C | 500 | 500 | 0.08 |  |  |  |
| Yolk-shell S@void@PPY | 0.2 A g^−1^ | 200 | 650 | 0.22 | 98 | 2.79 | [7] |
| Yolk-shell Au@MCNSs/S | 0.1 C | 40 | 664 | 0.93 | 50 | 0.5 | [8] |
| Yolk-shell S@Co-N-C/CNTs | 1 C | 300 | 712 | - | 66 | **-** | [9] |
| Multi-yolk-shell Co_4_N@carbon | 0.5 C | 300 | 669 | 0.09 | 70 | 1.2 | [10] |
| Co@NC | 0.5 C | 200 | 570 | 0.17 | 70 | 2.5 | [11] |
| Yolk-shell Co@NCNTs/S | 1 C | 400 | 700 | 0.06 | 64 | 1.84 | [12] |
| Yolk-Shell ZnO@Co-Doped NiO/S | 1 C | 500 | 501 | 0.08 | 70 | 1.8 | [13] |
| SnO_2_/carbon-carbon core-shell microspheres | 0.1 A g^−1^ | 150 | 602 | 0.40 | 60 | - | [14] |
| Yolk-shell Co-VN@C | 0.5 C | 200 | 715 | - | 70 | 1.3-1.5 | [15] |
|  | 1 C | 300 | 600 | 0.21 |  |  |  |
| Yolk-shell rGO/VO_2_ | 1 C | 400 | 516 | 0.07 | 70 | 1.8 | [16] |
| Core-shell S@δ-MnO_2_ | 0.2 C | 200 | 905 | 0.15 | 71 | 1 | [17] |
|  | 1 C | 200 | 480 | 0.26 |  |  |  |
| Yolk-shell HPC@S-PANI | 1A g^−1^ | 200 | 650 | 0.15 | 65 | 2.1 | [18] |
| Yolk-shell γ-Al_2_O_3_@C/S | 0.5 C | 500 | 494 | - |  | 0.96 | [19] |
| Core-Shell TiC@p-TiO_2_@S | 0.5 C | 160 | 603 | - | 74 | 2.3 | [20] |
| Yolk-shell NiS_2_/C-S | 1 C | 200 | 446 | 0.20 | 70 | 1 | [21] |
| NiCo_2_S_4_ yolk-shell hollow spheres | 0.5 C | 500 | 318 | 0.07 | 70 | 1.1-1.5 | [22] |
| Hollow-in-hollow carbon sphere | 1 A g^−1^ | 300 | 780 | 0.09 | 49 | 1.8 | [23] |
| Double-layered core-shell carbon sphere | 0.2 C | 150 | 900 | 0.11 | 55 | 1.5 | [24] |
| Bi_2_S_3_/C yolk-shell | 0.2 C | 300 | 282 | - | - | 1.8-2.1 | [25] |
| Core-shell NDHC@CeS | 0.5 C | 250 | 813 | 0.04 | 72 | 2 | [26] |
| Hollow ZIF-67-S-PPY | 0.1 C | 200 | 353 | 0.34 | 54 | 0.5-1.2 | [27] |
| Core-shell S@PPY | 0.2 C | 200 | 538 | 0.20 | 49 | 0.7-0.9 | [28] |
| S/PPY | 0.1 C | 50 | 613 | - | 62 | 2 | [29] |
| CS@HPP/S | 1 C | 300 | 716 | 0.07 | 72 | 2.6 | [30] |
| G@ppy-por | 0.2 C | 50 | 800 | 0.3 | - | 1.2 | [31] |
| Co-Bi/rGO–S | 1 C | 500 | 758 | 0.05 | 72 | 9.5 | [32] |
| H-LDH/Co_9_S_8_ | 0.1 C | 1500 | 395 | 0.05 | 79 | 1.5-2 | [33] |
| Ni_0.1_Zn_0.1_Co_0.8_Se_2_–S | 0.5 C | 100 | 682 | 0.19 | 60 | 4.6 | [34] |
| SVPA | 4 C | 500 | 732 | 0.05 | 87 | 2 | [35] |
| **S-PANI@S@S-PANI-HNPs** | 0.2 C | 200 | 986 | 0.07 | 73 | 2.5 | **This work** |
|  |  | 500 | 886 | 0.04 |  |  |  |
|  | 0.5 C | 200 | 829 | 0.11 |  |  |  |
|  |  | 500 | 641 | 0.08 |  |  |  |
|  | 1 C | 200 | 635 | 0.12 |  |  |  |
|  |  | 500 | 505 | 0.08 |  |  |  |

**Table S2** Description of studied samples

| **Sample name** | **Description** | **Sample type** | **Graphic illustration** |
| --- | --- | --- | --- |
| **S-NPs** | Sulfur nanoparticles | Reference | 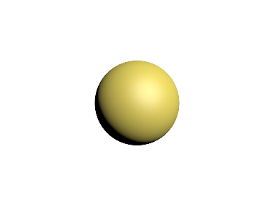 |
| **PANI-HNPs** | PANI hollow nanoparticles | Reference | 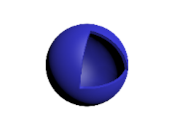 |
| **S@S-PANI-HNPs** | Sulfur coated PANI hollow nanoparticles with vulcanization | Reference | 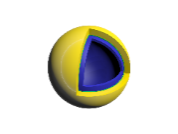 |
| **PANI@S@PANI-HNPs** | Sulfur hollow nanoparticles sandwiched in double PANI layers | Reference | 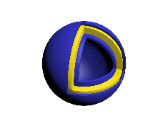 |
| **S-PANI@S@S-PANI-HNPs** | Sulfur hollow nanoparticles sandwiched in double PANI layers with vulcanization | Targeted sample | 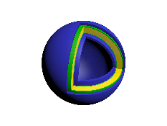 |

**Table S3** Binding energy of S 2p in PANI@S@PANI-HNPs and S-PANI@S@S-PANI-HNPs.

|  | **Peak 1**  **2p3/2** | **Peak 2**  **2p3/2** | **Peak 3**  **2p1/2** | **Peak 4**  **2p1/2** |
| --- | --- | --- | --- | --- |
| **PANI@S@PANI-HNPs** | 163.7 eV | 164.2 eV | 164.8 eV | 165.5 eV |
| **S-PANI@S@S-PANI-HNPs** | 163.5 eV | 164.0 eV | 164.7 eV | 165.3 eV |

**Table S4** Binding energy of N 1s in PANI@S@PANI-HNPs and S-PANI@S@S-PANI-HNPs.

|  | **Peak 1**  **‒N=** | **Peak 2**  **‒NH‒** | **Peak 3**  **‒N^+^‒** |
| --- | --- | --- | --- |
| **PANI@S@PANI-HNPs** | 398.9 eV | 399.5 eV | 400.3 eV |
| **S-PANI@S@S-PANI-HNPs** | 398.6 eV | 399.4 eV | 400.2 eV |

**Table S5** Binding energy of C 1s in PANI@S@PANI-HNPs and S-PANI@S@S-PANI-HNPs.

|  | **Peak 1**  **C‒C** | **Peak 2**  **C‒N** | **Peak 3**  **C=N** | **Peak 4**  π–π* |
| --- | --- | --- | --- | --- |
| **PANI@S@PANI-HNPs** | 283.9 eV | 284.6 eV | 285.5 eV | 287.0 eV |
| **S-PANI@S@S-PANI-HNPs** | 284.0 eV | 284.8 eV | 285.2 eV | 285.9 eV |

**References**

1. Zhou W, Yu Y, Chen H *et al.* Yolk-shell structure of polyaniline-coated sulfur for lithium-sulfur batteries. *J Am Chem Soc* 2013; **135**: 16736-43.

2. Pang Q, Nazar LF. Long-life and high-areal-capacity Li-S batteries enabled by a light-weight polar host with intrinsic polysulfide adsorption. *ACS Nano* 2016; **10**: 4111-8.

3. Li Z, Zhang J, Guan B *et al.* A sulfur host based on titanium monoxide@carbon hollow spheres for advanced lithium–sulfur batteries. *Nat Commun* 2016; **7**: 13065.

4. Zhang J, Shi Y, Ding Y *et al.* In situ reactive synthesis of polypyrrole-MnO_2_ coaxial nanotubes as sulfur hosts for high-performance lithium-sulfur battery. *Nano Lett* 2016; **16**: 7276-81.

5. Zhang J, Li Z, Chen Y *et al.* Ni-Fe layered double hydroxide hollow polyhedrons as a superior sulfur host for Li-S batteries. *Angew Chem Int Ed* 2018; **57**: 10944-8.

6. Ren Y, Hu J, Zhong H *et al.* Multiple core-shelled sulfur composite based on spherical double-layered hollow carbon and PEDOT:PSS as cathode for lithium–sulfur batteries. *J Alloys Compd* 2020; **837**: 155498.

7. Zhang M, Zhu M, Zhong Y *et al.* A novel sulfur@void@hydrogel yolk-shell particle with a high sulfur content for volume-accommodable and polysulfide-adsorptive lithium-sulfur battery cathodes. *Nanotechnology* 2020; **31**: 455402.

8. Zhang W, Yang C, Ding B *et al.* A self-crosslinking procedure to construct yolk-shell Au@microporous carbon nanospheres for lithium-sulfur batteries. *Chem Commun* 2020; **56**: 1215-8.

9. Liu R, Kang Q, Liu W *et al.* Carbon nanotube-connected yolk–shell carbon nanopolyhedras with cobalt and nitrogen doping as sulfur immobilizers for high-performance lithium–sulfur batteries. *ACS Applied Energy Materials* 2018; **1**: 6487-96.

10. Chen T, Kong W, Fan M *et al.* Chelation-assisted formation of multi-yolk–shell Co_4_N@carbon nanoboxes for self-discharge-suppressed high-performance Li–SeS_2_ batteries. *J Mater Chem A* 2019; **7**: 20302-9.

11. Faheem M, Li W, Ahmad N *et al.* Chickpea derived Co nanocrystal encapsulated in 3D nitrogen-doped mesoporous carbon: Pressure cooking synthetic strategy and its application in lithium-sulfur batteries. *J Colloid Interface Sci* 2021; **585**: 328-36.

12. Park S-K, Lee J-K, Kang YC. Yolk-shell structured assembly of bamboo-like nitrogen-doped carbon nanotubes embedded with Co nanocrystals and their application as cathode material for Li-S batteries. *Adv Funct Mater* 2018; **28**: 1705264.

13. Xu P, Liu H, Zeng Q *et al.* Yolk-shell nano ZnO@Co-doped NiO with efficient polarization adsorption and catalysis performance for superior lithium-sulfur batteries. *Small* 2021; **17**: e2005227.

14. Hong YJ, Lee J-K, Chan Kang Y. Yolk–shell carbon microspheres with controlled yolk and void volumes and shell thickness and their application as a cathode material for Li–S batteries. *J Mater Chem A* 2017; **5**: 988-95.

15. Ren W, Xu L, Zhu L *et al.* Cobalt-doped vanadium nitride yolk-shell nanospheres@carbon with physical and chemical synergistic effects for advanced Li-S batteries. *ACS Appl Mater Interfaces* 2018; **10**: 11642-51.

16. Song Z, Lu X, Hu Q *et al.* Construction of reduced graphene oxide wrapped yolk-shell vanadium dioxide sphere hybrid host for high-performance lithium-sulfur batteries. *Dalton Trans* 2020; **49**: 14921-30.

17. Li Q, Ma Z, Li J *et al.* Core-shell-structured sulfur cathode: Ultrathin delta-MnO_2_ nanosheets as the catalytic conversion shell for lithium polysulfides in high sulfur content lithium-sulfur batteries. *ACS Appl Mater Interfaces* 2020; **12**: 35049-57.

18. Wu F, Zhao S, Chen L *et al.* Electron bridging structure glued yolk-shell hierarchical porous carbon/sulfur composite for high performance Li-S batteries. *Electrochim Acta* 2018; **292**: 199-207.

19. Wu Y, Xiao Q, Huang S *et al.* Facile synthesis of hierarchically γ-Al_2_O_3_@C yolk-shell microspheres for lithium-sulfur batteries. *Mater Chem Phys* 2019; **221**: 258-62.

20. Zhang X, Yuan W, Yang Y *et al.* Immobilizing polysulfide by in situ topochemical oxidation derivative TiC@carbon-included TiO_2_ core-shell sulfur hosts for advanced lithium-sulfur batteries. *Small* 2020; **16**: e2005998.

21. Tian Y, Huang H, Liu G *et al.* Metal-organic framework derived yolk-shell NiS_2_/carbon spheres for lithium-sulfur batteries with enhanced polysulfide redox kinetics. *Chem Commun* 2019; **55**: 3243-6.

22. Tan X, Wang X, Wang X *et al.* NiCo_2_S_4_ yolk-shell hollow spheres with physical and chemical interaction toward polysulfides for advanced lithium-sulfur batteries. *Ionics* 2019; **25**: 4047-56.

23. Zang J, An T, Dong Y *et al.* Hollow-in-hollow carbon spheres with hollow foam-like cores for lithium–sulfur batteries. *Nano Research* 2015; **8**: 2663-75.

24. Zhou W, Xiao X, Cai M *et al.* Polydopamine-coated, nitrogen-doped, hollow carbon-sulfur double-layered core-shell structure for improving lithium-sulfur batteries. *Nano Lett* 2014; **14**: 5250-6.

25. Kim H, Kim D, Lee Y *et al.* Synthesis of Bi_2_S_3_/C yolk-shell composite based on sulfur impregnation for efficient sodium storage. *Chem Eng J* 2020; **383**: 123094.

26. Yan R, Oschatz M, Wu F. Towards stable lithium-sulfur battery cathodes by combining physical and chemical confinement of polysulfides in core-shell structured nitrogen-doped carbons. *Carbon* 2020; **161**: 162-8.

27. Geng P, Cao S, Guo X *et al.* Polypyrrole coated hollow metal–organic framework composites for lithium–sulfur batteries. *J Mater Chem A* 2019; **7**: 19465-70.

28. Zhou X, Chen F, Yang J. Core@shell sulfur@polypyrrole nanoparticles sandwiched in graphene sheets as cathode for lithium–sulfur batteries. *J Energy Chem* 2015; **24**: 448-55.

29. Yuan G, Wang H. Facile synthesis and performance of polypyrrole-coated sulfur nanocomposite as cathode materials for lithium/sulfur batteries. *J Energy Chem* 2014; **23**: 657-61.

30. Shaibani M, Mirshekarloo MS, Singh R *et al.* Expansion-tolerant architectures for stable cycling of ultrahigh-loading sulfur cathodes in lithium-sulfur batteries. *Sci Adv* 2020; **6**: eaay2757.

31. Zhao CX, Li XY, Zhao M *et al.* Semi-immobilized molecular electrocatalysts for high-performance lithium-sulfur batteries. *J Am Chem Soc* 2021; **143**: 19865-72.

32. Li C, Qi S, Zhu L *et al.* Regulating polysulfide intermediates by ultrathin Co-Bi nanosheet electrocatalyst in lithium−sulfur batteries. *Nano Today* 2021; **40**: 101246.

33. Chen S, Luo J, Li N *et al.* Multifunctional LDH/Co_9_S_8_ heterostructure nanocages as high-performance lithium–sulfur battery cathodes with ultralong lifespan. *Energy Storage Mater* 2020; **30**: 187-95.

34. Chen L, Xu Y, Cao G *et al.* Bifunctional catalytic effect of CoSe_2_ for lithium–sulfur batteries: single doping versus dual doping. *Adv Funct Mater* 2021; **32**: 2107838.

35. Kang H, Park MJ. Thirty-minute synthesis of hierarchically ordered sulfur particles enables high-energy, flexible lithium-sulfur batteries. *Nano Energy* 2021; **89**: 106459.
